# Heterologous Expression of *Panax ginseng PgTIP1* Confers Enhanced Salt Tolerance of Soybean Cotyledon Hairy Roots, Composite, and Whole Plants

**DOI:** 10.3389/fpls.2017.01232

**Published:** 2017-07-17

**Authors:** Jing An, Zhenmin Hu, Benning Che, Haiying Chen, Bingjun Yu, Weiming Cai

**Affiliations:** ^1^Laboratory of Plant Stress Biology, College of Life Sciences, Nanjing Agricultural University Nanjing, China; ^2^Institute of Plant Physiology and Ecology, Shanghai Institutes for Biological Sciences, University of Chinese Academy of Sciences, Chinese Academy of Sciences Shanghai, China

**Keywords:** *PgTIP1* transformation, salt tolerance, soybean hairy roots, transgenic soybean, water relations, ion homeostasis, ROS scavenging

## Abstract

The *Panax ginseng TIP* gene *PgTIP1* was previously demonstrated to have high water channel activity by its heterologous expression in *Xenopus laevis* oocytes and in yeast; it also plays a significant role in growth of *PgTIP1-*transgenic *Arabidopsis* plants under favorable conditions and has enhanced tolerance toward salt and drought treatment. In this work, we first investigated the physiological effects of heterologous *PgTIP1* expression in soybean cotyledon hairy roots or composite plants mediated by *Agrobacterium rhizogenes* toward enhanced salt tolerance. The *PgTIP1-*transgenic soybean plants mediated by the pollen tube pathway, represented by the lines N and J11, were analyzed at the physiological and molecular levels for enhanced salt tolerance. The results showed that in terms of root-specific heterologous expression, the *PgTIP1*-transformed soybean cotyledon hairy roots or composite plants displayed superior salt tolerance compared to the empty vector-transformed ones according to the mitigatory effects of hairy root growth reduction, drop in leaf RWC, and rise in REL under salt stress. Additionally, declines in K^+^ content, increases in Na^+^ content and Na^+^/K^+^ ratios in the hairy roots, stems, or leaves were effectively alleviated by *PgTIP1-*transformation, particularly the stems and leaves of composite soybean plants. At the whole plant level, *PgTIP1*-trasgenic soybean lines were found to possess stronger root vigor, reduced root and leaf cell membrane damage, increased SOD, POD, CAT, and APX activities, steadily increased leaf Tr, RWC, and Pn values, and smaller declines in chlorophyll and carotenoid content when exposed to salt stress compared to wild type. Moreover, the distribution patterns of Na^+^, K^+^, and Cl^-^ in the roots, stems, and leaves of salt-stressed transgenic plants were readjusted, in that the absorbed Na^+^ and Cl^-^ were mainly restricted to the roots to reduce their transport to the shoots, and the transport of root-absorbed K^+^ to the shoots was simultaneously promoted. *PgTIP1* transformation into soybean plants enhanced the expression of some stress-related genes (*GmPOD*, *GmAPX1*, *GmSOS1*, and *GmCLC1*) in the roots and leaves under salt treatment. This indicates that the causes of enhanced salt tolerance of heterologous *PgTIP1-*transformed soybean are associated with the positive regulation on water relations, ion homeostasis, and ROS scavenging under salt stress both at root-specific and whole plant levels.

## Introduction

Aquaporins (AQPs) are integral or intrinsic channel proteins with molecular weights of 21 to 34 kD and a conserved structure. They locate in the plasma and intracellular membranes of plant cells, where they facilitate the transport of water and/or a wide range of small neutral or uncharged solutes (glycerol, urea, boric acid, silicic acid, arsenite, ammonia, CO_2_, and H_2_O_2_, etc.) (Katsuhara et al., 2008; Maurel et al., 2008; Chaumont and Tyerman, 2014; Srivastava et al., 2014; Tian et al., 2016; Wang et al., 2016). Based on sequence homology or similarity, subcellular localization, and expression patterns, plant AQPs can generally be classified into five subfamilies: plasma membrane intrinsic proteins (PIPs), tonoplast intrinsic proteins (TIPs), nodulin26-like intrinsic proteins (NIPs), small and basic intrinsic proteins (SIPs), and uncategorized X intrinsic proteins (XIPs) (Kaldenhoff and Fischer, 2006; Danielson and Johanson, 2008). AQPs (mostly PIPs and TIPs) can provide plants with the means to rapidly and reversibly modify water permeability across cell membranes, and play a crucial role in plant water relations or homeostasis during plant growth, development, and even stress adaptation through the regulation of root water uptake or transport, leaf transpiration, or loss mediated by root and leaf hydraulic conductivity (Maurel et al., 2008; Chaumont and Tyerman, 2014). Furthermore, there is communication between apoplastic and intracellular reactive oxygen species (ROS) pools through certain AQPs (Dietz et al., 2016). For example, H_2_O_2_ is a relatively stable component of ROS compared with other ROS molecules, such as the superoxide anion radical ($O_{2}^{•-}$), and the production of H_2_O_2_ is typically apoplastic, resulting mainly from PM-located NADPH oxidase that catalyzes $O_{2}^{•-}$ formation which is then converted to H_2_O_2_ either spontaneously or by extracellular superoxidase (SOD). H_2_O_2_ can function as an important secondary messenger in signal transduction networks involved in the plant response under favorable and stressful environments (Saxena et al., 2016; Tian et al., 2016). Tian et al. (2016) suggested a pivotal role for an *Arabidopsis* aquaporin (AtPIP1;4) in the importing of extracellular H_2_O_2_ into the cytoplasm for apo-cytoplastic signal transduction and in activating responses for disease immunity or resistance.

At present, plants or crops often suffer from many abiotic (salt, drought, low temperature, heat, etc.) and biotic (disease, insects, weeds, etc.) stresses during their growth and development, and this appears to be growing in frequency as a result of global climate warming, severely restricting the development of modern sustainable agriculture (Rouached et al., 2015). Under a variety of adverse environments, AQPs may have positive (such as increasing root water uptake and reducing leaf water loss) or negative effects across different plant species, stress types, and intensity of the adversity. Additionally, the mechanisms by which AQPs regulate plant water relations or homeostasis are particularly complicated due to the diverse classes and functions for AQPs themselves. As a result of this, researchers often choose one or several particular interesting AQP members in a certain plant to conduct concentrated and systematic studies on the physiological and molecular functions, particularly focusing on adaptation to unfavorable conditions. Various biological analytical assays, such as *Xenopus laevis* oocytes, yeast expression systems, mesophyll protoplast, and even transgenic plants have been adopted for highlighting the beneficial functions of AQPs on plant water homeostasis and stress adaptation (Deshmukh et al., 2016). Many researches have now suggested that the differential (down- or up-regulated) transcriptional expression of AQP genes, especially for both PIPs and TIPs, and changes in AQP density through endomembrane trafficking and activity including posttranslational modifications, protein interaction, and subcellular relocalization are essential for plant water homeostasis, playing crucial roles in plant growth, development, and stress adaptation (Boursiac et al., 2005; Lin et al., 2007; Maurel et al., 2008; Wudick et al., 2009; Chaumont and Tyerman, 2014). A coordinated transcriptional down-regulation of certain *PIPs* and *TIPs* was found in *Arabidopsis* roots exposed to salt treatment, and may prevent deleterious effects from excessive salt uptake (Boursiac et al., 2005).

The *TIP* gene *PgTIP1*, which was found to be highly and specifically expressed in hormone-autotrophic ginseng cells, was demonstrated to have high water channel activity by its heterologous expression in *Xenopus laevis* oocytes with regards to the cell volume-changing rate, as well as in yeast by means of a protoplast bursting assay. It was also shown to have a significant role in the growth and development of *PgTIP1-*transgenic *Arabidopsis* plants under favorable conditions, including enhanced seed size and mass, and higher fatty acid content than the wild-type control (Lin et al., 2007; Li and Cai, 2015). Another related study by Peng et al. (2007) reported that the overexpression of *PgTIP1* in transgenic *Arabidopsis* plants led to enhanced tolerance against salt and drought stress, but lowered cold acclimation ability. Further work by Li and Cai (2015) suggested that the conferred faster growth and enhanced salt tolerance in transgenic *Arabidopsis* plants was related to its water channel activity of the Ser^128^ residue of *PgTIP1*. Functional analyses using the plant *PgTIP1-*transgenic technique have predominantly focused on *Xenopus laevis* oocytes, yeast, or non-crop *Arabidopsis*, whereas related studies on crops have rarely been reported. Of these crops, soybean (*Glycine max* L.) is the world’s leading economic oilseed crop and is also a major source of human food and animal feed (Manavalan et al., 2009). Although soybean is classified as a moderately NaCl-tolerant plant, the conventional breeding of soybean cultivars for improved salt tolerance is challenging due to its narrow basis of genetic germplasm and relatively limited salt tolerance (Li et al., 2006; Zhang et al., 2011). To date, about 70 AQP members have been discovered across the entire soybean genome, of which 23 are TIPs (Zhang et al., 2013; Deshmukh et al., 2016; Song et al., 2016). Wang et al. (2011) reported *Arabidopsis* plants overexpressing *GsTIP2;1* from *G. soja* displayed reduced tolerance to salt and drought stress when dehydration speed or water loss in the plant was enhanced. Song et al. (2016) studied the solute transport function of two soybean *TIPs* (*GmTIP1;5* and *GmTIP2;5*) by expression in *Xenopus laevis* oocytes, and found that GmTIP1;5 facilitated the rapid movement of water across the oocyte membrane, while GmTIP2;5 facilitated the movement of water and boric acid. Thus, different types of *TIP*-encoded proteins in plants display diverse selectivity for substrate and various stress responses to salt and drought. Consequently, research on the effects and physiological and molecular mechanisms of *PgTIP1* expression in widely cultivated crops such as soybean to improve tolerance of saline conditions, as well as the exploration of *PgTIP1* heterologous expression for obtaining new transgenic plant germplasms and salt-tolerant crop varieties, seems very necessary. The theoretical potential and practical application should be explored.

In this work, we used the globally cultivated salt-tolerant soybean cultivar Lee68 as the experimental material and investigated the physiological enhancement toward salt tolerance of heterologous *PgTIP1* expression in soybean cotyledon hairy roots and composite plants mediated by *A. rhizogenes.* We then selected our local soybean cultivar Nannong 8831 as a test material and identified and screened *PgTIP1* transgenic plants mediated by the pollen tube pathway, and analyzed the physiological and molecular events in enhanced salt tolerance of the representative lines N and J11. The objective of this study was to systematically reveal the physiological and molecular mechanisms of enhanced salt tolerance of *PgTIP1*-expressing soybean on aspects of salt stress-caused cell membrane damage, water absorption, salty ion transport, antioxidant enzymes activity or ROS level, and plant growth and photosynthesis, and to provide a theoretical and practical basis for the further exploration of salt-tolerant gene resources to be used in the cultivation or breeding of salt-tolerant transgenic soybean and other crop germplasms.

## Materials and Methods

### Plant Materials

Seeds of *G. max* (L.) cv. Lee68 were used for *PgTIP1* transformation into cotyledon hairy roots and composite plants, cv. Nannong 8831 for *PgTIP1-*transgenic soybean lines, and subsequent salt tolerance tests.

### Construction of the Plant Expression Vector

Primer sequences designed specifically for amplifying *PgTIP1* ORF (753 bp) were: F: CCCAAGCTTATGCCGATTCACGAAATTGC; R: TCCCCCGGGCTAGTAATCAGCGACAGGCAA where the underlined nucleotides depict *Hind* III and *Sma* I sites. The cDNA from *Panax ginseng* was used as template for amplification of the *PgTIP1* coding region. Amplified PCR products were subcloned into a pMD-18T vector and sequenced (Sangon Biotech., Co. Ltd., China). The ORF of *PgTIP1* anchored in the cloning vector was isolated following digestion with *Hind* III and *Sma* I and cloned into the plant expression vector (pCAMBIA0390) using a double promoter (35S+pUbi) and NOS terminator.

### *PgTIP1* Transformation into Soybean Cotyledon Hairy Roots, Composite Plants, and Whole Plants

The pCAMBIA0390-*PgTIP1* was transformed into the K599 strain (*A. rhizogenes*) for the transformation of soybean (cv. Lee68) cotyledon hairy roots and composite plants, which was conducted as in our previous work (Wei et al., 2016). Additionally, the reconstructed plasmid pCAMBIA0390-*PgTIP1* from *Escherichia coli* DH5α was transformed into soybean wild type (WT) (cv. Nannong 8831) using the pollen-tube pathway method (Moore et al., 1996; Li and Wu, 2007) and advanced to transgenic soybean lines (T_3_ generation).

### Nested PCR Assay

The nested polymerase chain reaction (PCR) method (Ao et al., 2011) was adopted to detect the exogenous *PgTIP1* gene expression in two runs for the transformed soybean cotyledon hairy roots, composite and whole plants, respectively. The primer in the first PCR for a target product of 1,865 bp is as: F: 5′-CTTCTTCGCCCGCCGTAAT-3′, R: 5′-ATGGCTCGTGACTGCTGCTGA-3′. The primer in the second PCR for a target product of 469 bp is as: F: 5′-TCGGTAGGTACGAAGAGTTCCGCC-3′, R: 5′-TCGATGGCGGTTGCGTAAACGG-3′. PCR reactions were performed using a PCR-cycler (ABI 2720, Applied Biosystems China, Inc., Beijing), with the reaction mixture consisting of 10× rTaq buffer (2.5 μL), 2.5 mM dNTPs (2 μL), 25 mM MgCl_2_ (2 μL), 10 μM primer concentration (1 μL), 50 ng of prepared cDNA (1 μL), 5U/μL rTaq (0.2 μL), 16.3 μL of ddH_2_O in a final volume of 25 μL. The first PCR conditions were as follows: an initial denaturation (3 min, 94°C) followed by 35 cycles of denaturation (30 s, 94°C), annealing (50 s, 61°C) and extension (1 min and 50 s, 72°C), and an additional extension at 72°C for 10 min. The second PCR conditions were as follows: an initial denaturation (3 min, 94°C) followed by 35 cycles of denaturation (30 s, 94°C), annealing (30 s, 55°C) and extension (50 s, 72°C), and an additional extension at 72°C for 10 min. The PCR products were separated on 1.0% agarose gel and then visualized by Goldview staining on UV transilluminator.

### Salt Tolerance Test of *PgTIP1*-Transformed Soybean Cotyledon Hairy Roots

The hairy roots of the *PgTIP1-*transformed soybean cotyledons were obtained according to the methods of Wei et al. (2016) using pCAMBIA0390-*PgTIP1*-containing *A. rhizogenes* strain K599. New hairy roots that had sprouted from the infected cotyledons that were free of *Agrobacterium*, as screened by nested PCR, and at similar lengths were selected and transferred into ½ Hoagland solution containing 0 and 80 mM NaCl. Hairy roots infected with *A. rhizogenes* strain K599 containing the empty vector pCAMBIA0390 served as the control. After 5 days of treatment, the root growth was photographed, the maximal root length was quantified with a ruler, and the fresh or dry weights, and extraction and contents of Na^+^ and K^+^ were measured as indicated previously (Wei et al., 2015).

### Salt Tolerance Test of *PgTIP1-*Transformed Soybean Hairy Root Composite Plants

*PgTIP1-*transformed soybean (cv. Lee68) hairy root composite plants were obtained according to the methods of Wei et al. (2016) by infection with *A. rhizogenes* strain K599 containing the pCAMBIA0390-*PgTIP1*. Hairy roots that were free of *Agrobacterium*, as screened by nested PCR, were selected and the original roots removed from the seedlings. Seedlings infected with *A. rhizogenes* strain K599 containing the empty vector pCAMBIA0390 served as the negative control. These seedlings were cultured in ½ Hoagland solution for 10 days at 18 ± 2°C to 25 ± 2°C and a 14 h/10 h (day/night) photoperiod. The seedlings with hairy roots of a similar-length were selected and treated with ½ Hoagland solution (pH = 6.5) containing 120 mM NaCl for 5 days, and seedlings grown in ½ Hoagland solution without NaCl served as the untreated control. During this period, the solutions listed above were replaced once. After 5 days of treatment, the seedlings were photographed, and the fresh weight of the roots, relative water content (RWC), and relative electrolytic leakage (REL) of the 1st pair of unifoliolate leaves, and contents of Na^+^, K^+^ of the roots, stems, and whole leaves were measured accordingly. Values of RWC and REL were determined using our previous methods (Hu et al., 2016). Extraction and assaying of Na^+^ and K^+^ were also performed as aforementioned.

### Salt Tolerance Assay on *PgTIP1-*Transgenic Soybean Plants

Seeds (T_3_ generation) of *PgTIP1* transgenic soybean plants (lines N and J11) and WT (cv. Nannong 8831) were screened by nested PCR and surface-sterilized with 1 g⋅L^-1^ HgCl_2_ for 5 min, fully rinsed in distilled water, soaked in distilled water for 10 h, and germinated at 25°C in the dark. The germinated seeds (each with 0.5∼1 cm epicotyl) were planted in plastic pots (25 × 18 cm) containing quartz sand, fertigated with ½ Hoagland solution, and maintained in a greenhouse with a temperature ranging from 18 ± 2°C to 25 ± 2°C and a 14 h/10 h (day/night) photoperiod. At the time of the 1st trifoliate leaf expansion, the plants were subjected to salt stress by fertigating with ½ Hoagland solution plus 150 mM NaCl, or ½ Hoagland solution devoid of NaCl as the control treatment. The Hoagland solution treatments were replaced every 2 days.

Seven days later, the seedlings were photographed and sampled to measure the RWC of the leaves, REL of the roots and leaves, and Na^+^, K^+^ contents in the roots, stems, and leaves using the methods described earlier. Root vigor was determined according to the triphenyltetrazolium chloride (TTC) method (Wang et al., 2012). Chlorophyll and carotenoid contents in the leaves were determined using a UV-9100 spectrophotometer (Beijing, China) following the procedures of Qu et al. (2009). Cl^-^ contents in the roots, stems, and whole leaves were assayed using the method of Zhou and Yu (2009). Net photosynthetic rate (Pn) and transpiration rate (Tr) of the leaves was determined by a portable photosynthesis meter (LI-6400XT, LI-COR Inc., United States). Photosynthetic photon flux density (PPFD), temperature, and CO_2_ concentration were 1,000 μmol m^-2^ s^-1^, 28°C, and 382∼385 μmol mol^-1^, respectively. The maximum photochemical efficiency of PSII (*F*v/*F*m) was measured at room temperature using a plant efficiency analyzer (Handy PEA Fluorometer, Hansatech Instruments, United Kingdom). The flux density of incident photosynthetically active radiation (PAR) was 3,000 μmol m^-2^ s^-1^. The leaf blades were dark-adapted for 30 min using Handy-PEA leaf clips and the *F*v/*F*m values were read directly (Tian et al., 2014).

The production rate of $O_{2}^{•-}$ was measured by the modified method of Elstner and Heupel (1976). Briefly, fresh tissues (1.0 g) were homogenized in 2 mL of 50 mM phosphate buffer (pH 7.8) and then centrifuged at 10,000 × *g* for 10 min. The supernatants (1 mL) were mixed with 0.9 mL of 50 mM phosphate buffer (pH 7.8) and 0.1 mL of 10 mM hydroxylamine chlorohydrate, and then incubated at 25°C. After 30 min, 1 mL of the above-mentioned culture solution was added to 1 mL of 17 mM sulfanilamide and 1 mL 7 mM α-naphthylamine at 25°C for 20 min. The absorbance was measured at 530 nm and $O_{2}^{•-}$ production rate was calculated from a standard curve of NaNO_2_. H_2_O_2_ content was measured following the method of Sergiev et al. (1997). Briefly, the roots or leaves (0.2 g) were homogenized with 2 mL of 0.1% (w/v) trichloroacetic acid (TCA) in an ice bath and then centrifuged at 12,000 × *g* for 15 min at 4°C. Then, 0.5 mL of the supernatant was added to 0.5 mL 10 mM phosphate buffer (pH 7.0) and 1 mL 1 M KI. The absorbance of the mixture was read at 390 nm. Finally, the content of H_2_O_2_ was calculated from a standard curve. The antioxidant enzyme extractions were performed according to the method of our previous work (Zhao et al., 2017), the roots or leaves (0.2 g) were homogenized in a mortar and pestle with 2 mL of 50 mM ice-cold phosphate buffer (pH 7.0) containing 1 mM EDTA⋅Na_2_ and 0.5% PVP (w/v). The homogenate was centrifuged at 15,000 × *g* for 15 min at 4°C. The supernatant was used as the enzyme extract for assays of SOD, POD, CAT, and APX activities. SOD activity was assayed using the photochemical nitroblue tetrazolium (NBT) method (Beauchamp and Fridovich, 1971). The reaction mixture contained 100 mM phosphate buffer (pH 7.8), 130 mM methionine, 750 μM NBT, 20 μM riboflavin, 1 mM EDTA⋅Na_2_, deionized water, and 80 μL enzyme extracts in a 3 mL volume. One unit of SOD activity was defined as the amount of enzyme required to cause 50% inhibition of NBT reduction monitored at 560 nm. POD activity was measured with guaiacol as the substrate according to Nakano and Asada (1981). The reaction mixture (3 mL) consisted of 100 mM sodium acetate buffer (pH 5.4), 10 mM guaiacol solution, 0.1 mM H_2_O_2_ and 10 μL enzyme extracts. The increase in absorbance due to oxidation of guaiacol was measured at 470 nm for 1 min. One unit of POD activity was defined as a change in absorbance of 0.1 unit⋅min^-1^. CAT activity was assayed according to Aebi (1984). The 3-mL reaction mixture contained 0.1 mL of enzyme extracts, 0.1 M phosphate buffer (pH 7.0), deionized water and 20 mM H_2_O_2_. The decomposition of H_2_O_2_ was measured by following the decrease in absorbance at 240 nm for 3 min and quantified by its molar extinction coefficient (39.4 mmol⋅L^-1^⋅cm^-1^). One unit of CAT activity was defined as a change in absorbance of 0.1 units min^-1^ caused by addition of the enzyme extracts. APX activity was assayed following the method of Nakano and Asada (1981). The reaction mixture of 3 mL contained 100 mM phosphate (pH 7.0), 100 mM ascorbic acid, 0.1 mM H_2_O_2_, deionized water and 0.1 mL enzyme extracts. The reaction was started by adding enzyme extracts to the mixture. Enzyme activity was quantified by following the decrease in absorbance at 290 nm for 3 min. One unit of APX activity was defined as an absorbance change of 0.01 units⋅min^-1^.

For semi-quantitative RT-PCR analysis of the stress-related genes expression, total RNA was isolated from the roots and leaves of soybean cv. Nannong 8831 and *PgTIP1-*transgenic lines N and J11 using a MiniBEST Plant RNA Extraction Kit (TaKaRa) according the manufacturer’s instructions. The RNA was stored at -80°C and total RNA was quantified using a NanoDrop spectrophotometer. First-strand cDNAs were synthesized from total RNA using a PrimeScript RT reagent kit (TaKaRa). Semi-quantitative RT-PCR was performed at least three independent replicates using a pair of gene-specific primers. The housekeeping gene *GmActin* was used as an internal control. The amplification program for this work was performed at 94°C for 5 min, followed by 28 cycles of 94°C for 30 s, 53°C for 30 s, 72°C for 50 s, and a final extension of 72°C for 10 min. All primers used for RT-PCR are listed in **Table 1**. Note: in all experiments for *PgTIP1*-transgenic soybean lines, the 1st pair of unifoliolate leaves were used for the assays, with the exception of measurements of leaf Na^+^, K^+^, and Cl^-^ contents using whole leaves (including the 1st pair of unifoliolate, the 1st and 2nd trifoliate leaves).

**Table 1:** Primers for RT-PCR of the stress-related genes.

Gene names	Accession no.		Primer sequences (5′ → 3′)
*GmTIP1;1*	AK285481	F	TGGAGTCGGAGTTGGCAAC
		R	GTGTGGCTGATGAAGACAACC
*GmTIP1;3*	NM_001255778	F	TCGTCTACGTCATCGCC
		R	AACCTCGTAGATAAGCCCAG
*GmSOD1*	NM_001248369	F	CGTAACTGGATCTCTTGCTG
		R	CAGAATCAGCATGGACAACA
*GmCAT1*	NM_001250627	F	AAGTGTGCCCATCACAACAATC
		R	AGAACGATCAGCCTGAGACC
*GmPOD*	NM_001251386	F	TGCTTTGTTCAAGGTTGTGA
		R	CTCAGGTCCAAATTGGTGAG
*GmAPX1*	NM_001250856	F	TGGACCTGAAGTTCCATTCCAC
		R	AAGAAGGCATCTTCGTCCG
*GmNHX1*	AY972078	F	GGGGCACACTTCACTAAGA
		R	CCATTACGTTCAGTTGGTGA
*GmSOS1*	NM_001258010	F	TTACACTACCTTGGCATGGA
		R	CAGTCACATAGAGGCTCAGA
*GmCLC1*	AY972079	F	CCATCATTCTCATGGGTTCC
		R	ACTTGGATAAGGTGTGCTCT
*GmActin*	V00450	F	GTTCTCTCCTTGTATGCAAGTG
		R	CCAGACTCATCATATTCACCTTTAG


### Statistical Analyses

All data were analyzed and presented as means ± SD for each treatment (*n* = 3; or *n* = 10 for fresh weight or maximal length of hairy roots) using SPSS software ver. 20.0, and the mean differences were assessed using Duncan’s test (*P* < 0.05).

## Results

### *PgTIP1* Mitigates NaCl Stress on Transformed Soybean Cotyledon Hairy Roots by Reducing Na^+^ Accumulation and Na^+^/K^+^ Ratio

**FIGURE 1:**
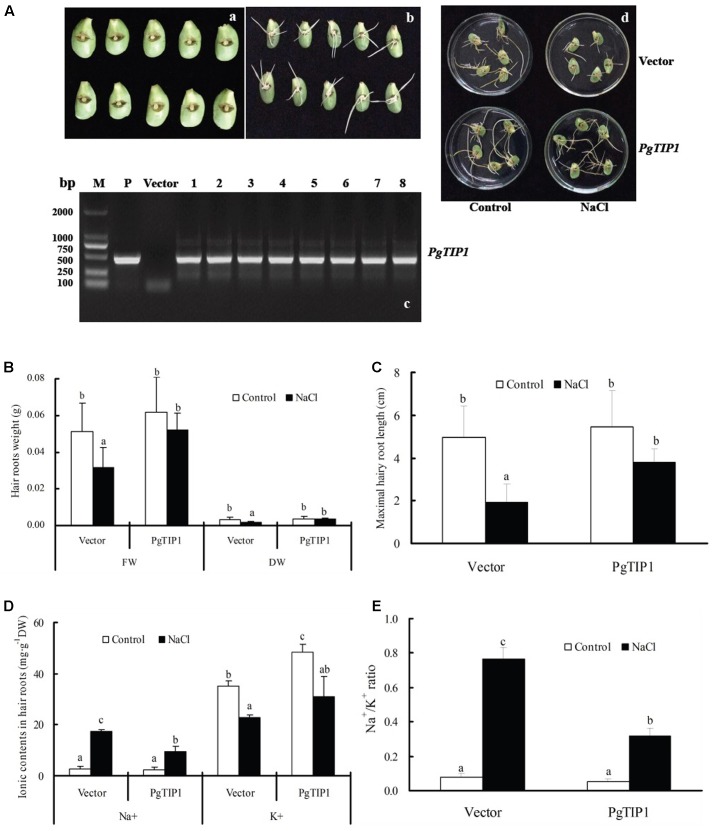
Growth characteristics of hairy roots of *PgTIP1-*transformed soybean cotyledons under salt stress. (**A-a,b**) Culture of hairy roots of *PgTIP1-*transformed soybean cotyledons. **(A-c)** PCR verification, **(A-d)** changes in growth phenotype, **(B)** FW and DW, **(C)** maximal hairy root length, **(D)** contents of Na^+^ and K^+^, and **(E)** Na^+^/K^+^ ratio in the hairy roots under 80 mM NaCl treatment for 5 days. Each bar represents as means ± SD for each treatment (*n* = 3; or *n* = 10 for fresh weight or maximal length of hairy roots), means in bars followed by different letters show significant difference (*P* < 0.05) between the parameters of the empty vector-transformed and *PgTIP1-*transfromed soybean cotyledon hairy roots.

Using the *A. tumefaciens* K599-mediated hairy roots system, the empty vector-transformed and *PgTIP1-*transformed soybean (cv. Lee68) cotyledon hairy roots were obtained and the positive lines were identified by nested PCR (**Figures 1A-a,b,c**). When the empty vector-transformed and *PgTIP1-*transformed soybean cotyledon hairy roots were exposed to 80 mM NaCl solution for 5 days, root growth was clearly inhibited in comparison with the non-treated ones, especially the empty vector-transformed cotyledon hair roots (**Figure 1A-d**). This phenomenon is also reflected in the decreasing fresh or dry root weight (**Figure 1B**) and maximal root length (**Figure 1C**) extents under salt treatment. With respect to Na^+^ and K^+^ contents, salt stress significantly increased Na^+^ content and reduced K^+^ content, accordingly resulting in a large rise in the Na^+^/K^+^ ratio in the empty vector-transformed and *PgTIP1-*transformed soybean cotyledon hairy roots. Notably, the increasing ranges of Na^+^ content and Na^+^/K^+^ ratio in the empty vector-transformed soybean cotyledon hairy roots were remarkably higher than those of the *PgTIP1-*transformed (**Figures 1D,E**).

### *PgTIP1* Alleviates Salt Injury on Transformed Soybean Hairy Root Composite Plants by Adjusting Water Relations, Na^+^ and K^+^ Levels

**FIGURE 2:**
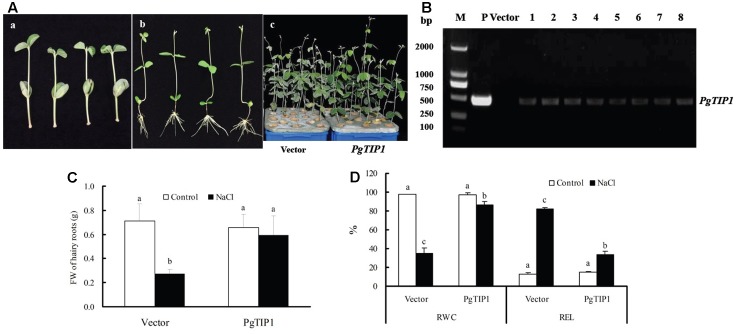
Growth characteristics of *PgTIP1-*transformed soybean hairy root composite plants under salt stress. **(A-a,b)** Culture of *PgTIP1-*transformed soybean hairy root composite plants, **(B)** PCR verification, **(A-c)** changes in growth phenotype, **(C)** FW of hairy roots, and **(D)** RWC and REL of the 1st pair of unifoliolate leaves under 120 mM NaCl treatment for 5 days. Each bar represents as means ± SD for each treatment (*n* = 3), means in bars followed by different letters show significant difference (*P* < 0.05) between the parameters of the empty vector-transformed and *PgTIP1-*transfromed soybean hairy root composite plants.

When the 1st pair of unifoliolate leaves had fully expanded, the normally cultivated soybean (cv. Lee68) seedlings were pruned from the original roots and infected by immersing the cotyledon hypocotyls with empty vector-contained and *PgTIP1-*contained *A. tumefaciens* K599. After approximately 20 days of culture, soybean hairy root composite plants were eventually formed (**Figures 2A-a,b**). Then, 8 to 10 seedlings were randomly selected for *PgTIP1*-transformation verification by nested PCR, and fine transformation efficiency was displayed (**Figure 2B**). When the empty vector-transformed and *PgTIP1-*transformed soybean hairy root composite plants were treated with 120 mM NaCl for 5 days, the leaves of the former showed obvious wilting, while the transgenic plants suffered much less (**Figure 2A-c**). The fresh weights (FW) of the hairy roots and the RWCs of the unifoliolate leaves of the empty vector-transformed plants were significantly decreased in comparison to the untreated plants, but the REL of unifoliolate leaves increased significantly (*P* < 0.05). The overall changes in *PgTIP1-*transformed composite plants were relatively smaller, and the decrease in FW of the hairy roots was not statistically significant (*P* > 0.05; **Figures 2C,D**).

**FIGURE 3:**
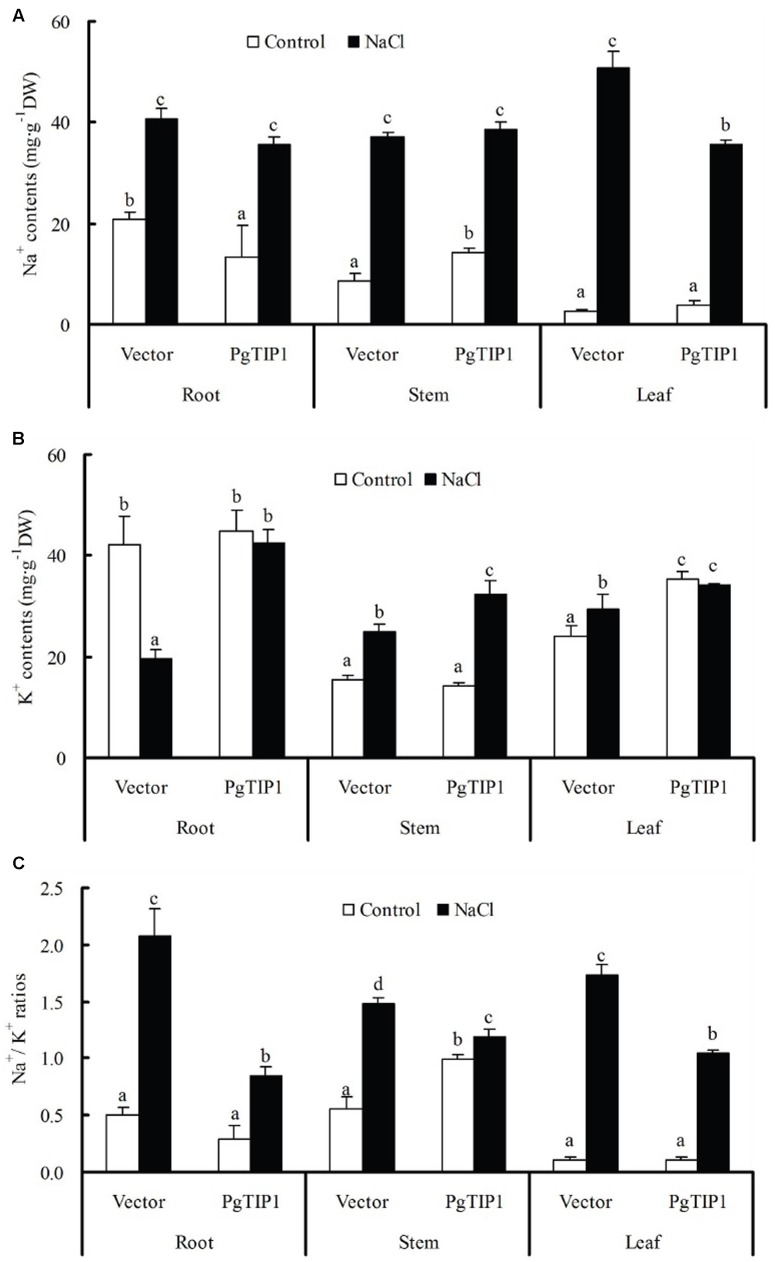
Changes in contents of **(A)** Na^+^, **(B)** K^+^, and **(C)** Na^+^/K^+^ ratios in roots, stems and leaves of the empty vector-transformed and *PgTIP1*-transformed soybean hairy root composite plants under salt stress. The seedlings with hairy roots of a similar-length were selected and treated with ½ Hoagland solution containing 120 mM NaCl for 5 days, and seedlings grown in ½ Hoagland solution without NaCl served as the untreated control. During this period, the solutions listed above were replaced once. The sampled leaves included the 1st pair of unifoliolate, the 1st and 2nd trifoliate leaves. Each bar represents the mean and SD of three replicates, means in bars followed by different letters show significant difference (*P* < 0.05) between the parameters of the empty vector-transformed and *PgTIP1-*transfromed soybean hairy root composite plants.

Under salt stress, the Na^+^ contents in the roots, stems, and leaves of empty vector-transformed and *PgTIP1*-transformed hairy root composite plants were significantly higher than those of untreated plants (*P* < 0.05). The increases in Na^+^ content in the roots and stems of the two types of soybean plants were similar, but the rise in Na^+^ content in the leaves of the *PgTIP1*-transformed composite plants was markedly lower than that of the empty vector-transformed plants (**Figure 3A**). As for K^+^ contents, the NaCl treatment led to a significant drop or slight decline in the roots of empty vector-transformed or *PgTIP1-*transformed composite plants, respectively, but K^+^ contents in the stems and leaves of both soybean plants showed an increasing trend, and those of *PgTIP1-*transformed plants were evidently higher than the empty vector-transformed plants (**Figure 3B**). Echoed by the changes in K^+^ and Na^+^ contents, the Na^+^/K^+^ ratios in the roots, stems, and leaves of both soybean plants were significantly increased under salt stress, and as a whole, the increases in *PgTIP1*-transformed plants were obviously lower than the empty vector-transformed plants (**Figure 3C**).

### *PgTIP1* Alleviates NaCl Stress on Transgenic Soybean Plants by Reducing Cell Membrane Damage, Improving Photosynthesis and Antioxidant Enzyme Activities, and Adjusting Ionic Distribution

**FIGURE 4:**
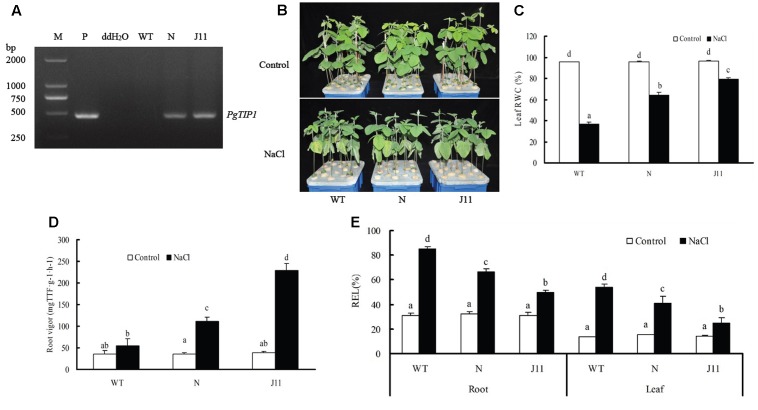
Growth phenotype and physiological responses of WT and *PgTIP1-*transgenic soybean lines under NaCl treatment. **(A)** The seedlings (T_3_ generation) of *PgTIP1* transgenic soybean lines N and J11, and WT (cv. Nannong 8831) were screened by PCR. The PCR-verified seeds and WT were surface-sterilized, soaked, germinated, and planted in plastic pots containing quartz sand, fertigated with ½ Hoagland solution, and maintained in a greenhouse with a temperature ranging from 18 ± 2°C to 25 ± 2°C and a 14 h/10 h (day/night) photoperiod. At the time of the 1st trifoliate leaf expansion, the plants were subjected to salt stress by fertigating with ½ Hoagland solution plus 150 mM NaCl, or ½ Hoagland solution devoid of NaCl as the control treatment. The Hoagland solution treatments were replaced every 2 days. After 7 days treatment, **(B)** the growth phenotype was photographed, and measured **(C)** RWC in the 1st pair of unifoliate leaves, **(D)** root vigor, and **(E)** REL in roots and the 1st pair of unifoliate leaves. Each bar represents the mean and SD of three replicates, means in bars followed by different letters show significant difference (*P* < 0.05) between the parameters of WT and *PgTIP1-*transgenic lines.

At the whole plant level, two *PgTIP1*-transgenic soybean lines N and J11 (cv. Nannong 8831 as WT) were identified by nested PCR (**Figure 4A**). Under favorable conditions, seedlings of WT and transgenic soybean lines all grew well. When subjected to 150 mM NaCl solution for 7 days, WT plants displayed obvious withering and yellowing of the leaves and lower RWC in the leaves, whereas the two *PgTIP1*-transgenic lines showed relatively mild salt injury symptoms and maintained relatively higher leaf RWC. The J11 line particularly appeared healthier (**Figures 4B,C**). Under salt stress, root vigor of WT and two transgenic lines were all enhanced with the exception of the NaCl-treated and untreated WT plants which showed no significant difference (*P* > 0.05). With regards to both transgenic-*PgTIP1* lines, J11 showed the highest root vigor in the NaCl-treatment (**Figure 4D**). When taking into consideration the REL values of salt-stressed roots and leaves, remarkable increases were observed in the three types of soybean material in comparison with each control, and the increases in lines N and J11 were visibly lower that WT, and J11 presented the smallest change (**Figure 4E**).

**FIGURE 5:**
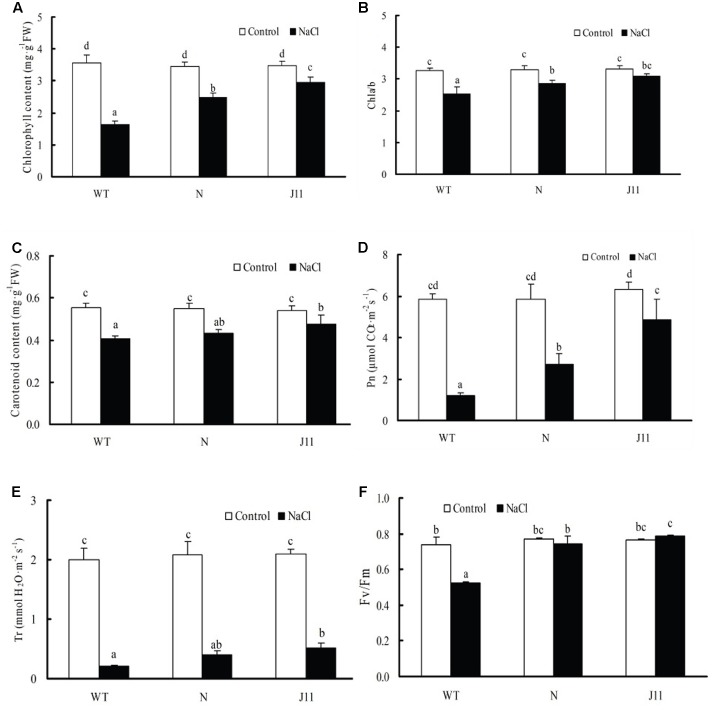
Changes in photosynthetic parameters in leaves of WT and *PgTIP1-*transgenic soybean lines under NaCl treatment. The plants of WT and *PgTIP1* transgenic soybean lines N and J11 were cultured and NaCl-treated as above-mentioned in **Figure 4**. After 7 days treatment, the 1st pair of unifoliate leaves were sampled for measuring **(A)** chlorophyll content, **(B)** chla/b, **(C)** carotenoids content, or used for measuring **(D)** Pn, **(E)** Tr, and **(F)** *F*v/*F*m in leaves of WT and *PgTIP1-*transgenic soybean lines. Each bar represents the mean and SD of three replicates, means in bars followed by different letters show significant difference (*P* < 0.05) between the parameters of WT and *PgTIP1-*transgenic lines.

Under 150 mM NaCl treatment for 7 days, the chlorophyll and carotenoid contents, chla/b ratios, and values of Pn and Tr in the leaves of WT and *PgTIP1*-transgenic lines N and J11 were all inferior to those of each control. Drops in these parameters in WT approached the significant level (*P* < 0.05), while declines in both the transgenic lines were relatively smaller. In particular, declines in the chla/b ratio of J11 showed no significant difference to untreated plants (*P* > 0.05), and its Tr value was even significantly higher than that of salt-stressed WT plants (**Figures 5A–E**). With respect to the maximum photochemical efficiency of PSII (*F*v/*F*m), it was evidently depressed (*P* < 0.05) in salt-stressed WT, but no significant changes in the transgenic lines N and the J11 were observed, and J11 even exhibited a slight increase (*P* > 0.05) (**Figure 5F**).

**FIGURE 6:**
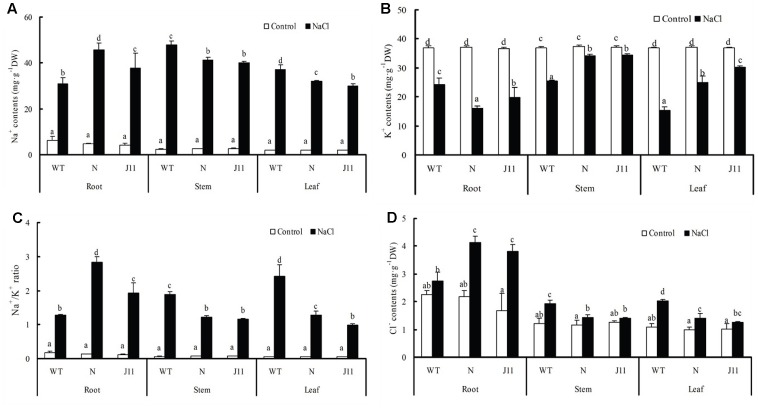
Changes in ion contents of plants of *PgTIP1-*transgenic soybean lines and WT under NaCl treatment. The plants of WT and *PgTIP1* transgenic soybean lines N and J11 were cultured and NaCl-treated as above-mentioned in **Figure 4**. After 7 days treatment, the roots, stems and leaves (including the 1st pair of unifoliolate, the 1st and 2nd trifoliate leaves) were sampled for measuring contents of **(A)** Na^+^ and **(B)** K^+^, **(C)** Na^+^/K^+^ ratios, and **(D)** Cl^-^ content. Each bar represents the mean and SD of three replicates, means in bars followed by different letters show significant difference (*P* < 0.05) between the parameters of WT and *PgTIP1-*transgenic lines.

Under salt stress, Na^+^ and Cl^-^ contents in the roots, stems, and leaves of WT and the transgenic lines N and J11 were obviously increased compared to each of the non-treated plants, and variations in Na^+^ level were particularly large. Conversely, the K^+^ contents in the roots, stems, and leaves of the three types of soybean seedlings were distinctly decreased, therefore leading to sharp increases in the Na^+^/K^+^ ratio. Interestingly, increases in the ranges of Na^+^ and Cl^-^ contents and Na^+^/K^+^ ratio, together with the lower ranges of K^+^ contents in the roots of NaCl-treated transgenic lines N and J11 were clearly greater than those in the roots of WT, but the opposite was observed for the shoots including the stems and leaves, and in particular, Cl^-^ content in the stems of transgenic line J11 did not differ significantly to the control (**Figure 6**).

**FIGURE 7:**
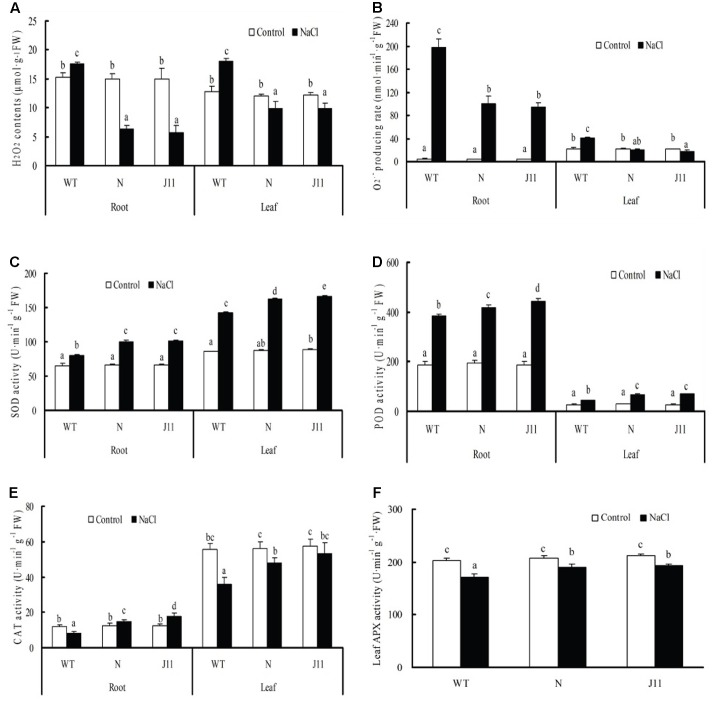
Changes in ROS levels and antioxidant enzyme activities of plants of *PgTIP1-*transgenic soybean lines and WT under NaCl treatment. The plants of WT and *PgTIP1* transgenic soybean lines N and J11 were cultured and NaCl-treated as above-mentioned in **Figure 4**. After 7 d treatment, the roots and the 1st pair of unifoliolate leaves were sampled for measuring **(A)** H_2_O_2_ content, **(B)** $O_{2}^{•-}$ producing rate, activities of **(C)** SOD, **(D)** POD, **(E)** CAT, and **(F)** APX. Each bar represents the mean and SD of three replicates, means in bars followed by different letters show significant difference (*P* < 0.05) between the parameters of WT and *PgTIP1-*transgenic lines.

Under NaCl treatment for 7 days, H_2_O_2_ content and $O_{2}^{•-}$ production rate in the roots and leaves of WT were significantly increased compared with the control, while for transgenic lines N and J11 the H_2_O_2_ content in the roots and leaves, and $O_{2}^{•-}$ production rate in the leaves, were obviously decreased in contrast with each control (**Figures 7A,B**), with the exception of the obvious rise in $O_{2}^{•-}$ production rate in the roots (but lower than in WT). Salt exposure resulted in enhanced SOD and POD activity in the roots and leaves of the three types of soybean plants, and the enhancing effects on transgenic lines N and J11, especially the latter, were pronounced (**Figures 7C,D**). Additionally, CAT activities in the roots of N and J11 were improved by salt stress in comparison to the control, whereas CAT activity in the roots and leaves of WT and the leaves of N and J11, and APX activity in the leaves of the three types of soybean decreased significantly, and WT showed the largest drop (**Figures 7E,F**).

### Transcriptional Patterns of Stress-Related Genes in the Roots and Leaves of NaCl-Treated *PgTIP*-Transgenic Soybean Plants

**FIGURE 8:**
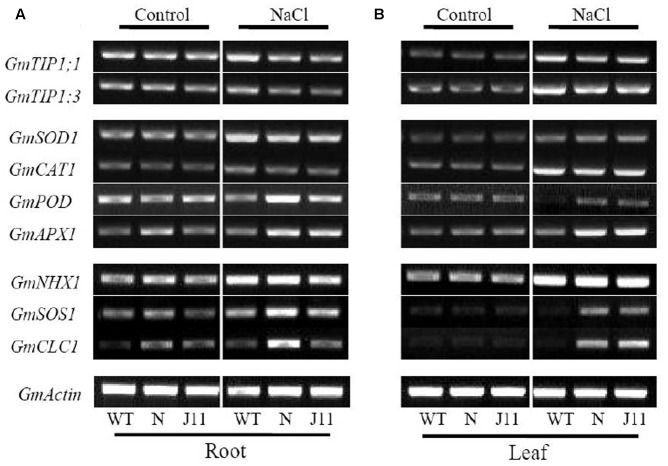
Transcriptional patterns of stress-related genes in plants of NaCl-treated WT and *PgTIP*-transgenic soybean lines. At the time of the 1st trifoliate leaf expansion, plants of *PgTIP1-*transgenic soybean lines N and J11, and WT were subjected to 150 mM NaCl treatment for 24 h, the genes expression patterns of *GmSOD1*, *GmCAT1*, *GmPOD*, *GmAPX1*, *GmNHX1*, *GmSOS1*, *GmCLC1*, *GmTIP1;1*, and *GmTIP1:3* in **(A)** roots and **(B)** leaves were analyzed by semi-quantitative RT-PCR using a pair of gene-specific primers (listed in **Table 1**), which was performed at least three independent replicates, and the representative one was showed. The housekeeping gene *GmActin* was used as an internal control.

Considering soybean endogenous stress-related genes as the pointcut, when seedlings of *PgTIP1*-transgenic lines N and J11, and WT, were exposed to 150 mM NaCl for 24 h, the transcriptional levels of *GmPOD* and *GmAPX1*, which partly contribute toward the maintenance of cellular ROS homeostasis, were obviously heightened in the roots and leaves of the two transgenic lines compared to WT. The expression levels of *GmSOD1* and *GmCAT1* in the roots and leaves displayed unobvious variation among the three. The expression of *GmSOS1* and *GmCLC1*, which are directly involved in cellular Na^+^ and Cl^-^ homeostasis, were also reinforced in the roots and leaves of N and J11, although the transcription of the *GmNHX1* gene, which is important for intracellular Na^+^ compartmentation, showed no difference in the roots and leaves under favorable or saline conditions. We also investigated two representative soybean *TIP* genes, *GmTIP1;1* and *GmTIP1;3* for the highest homology of *PgTIP1*, and found that both expressions were scarcely affected in the roots and were similarly enhanced in the leaves under salt treatment (**Figures 8A,B**).

## Discussion

Water deficit commonly damages plants suffering under various adverse conditions, and salt stress, ionic poison, nutritional imbalance, and ROS injury often also occur under such circumstances. The maintenance of water status is not only vital for the growth of plants under favorable conditions, but is also crucial for plant adaptation to salt, drought, and other abiotic stresses (Hu et al., 2016). Plant water relations and ionic homeostasis with reciprocal regulation are important key factors that contribute toward salt tolerance in plants. Plant AQPs play a crucial role in maintaining plant water homeostasis under normal or stressful environments, and among of them, PIPs, as the largest category, are located in the plasma membrane (PM) and generally control both water uptake through the roots and water loss via the leaves by the symplastic route through cytoplasmic continuities and plasmodesmata, and the transcellular path across cell plasma membranes (Maurel et al., 2008). At present, homologous overexpression or heterologous expression of one or more particular AQP gene has become a widely used strategy to uncover its functions in plant water relations or homeostasis under water deficit caused by salt or drought stress, and possibly presents a feasible approach for the development of transgenic crop cultivars with improved tolerance to abiotic and biotic stresses. The antisense *NtAQP1*-expressing tobacco plants showed more sensitivity to drought stress (Siefritz et al., 2002), and the over-expression of *NtAQP1* in tobacco plants displayed enhanced salt stress resistance in comparison with wild plants (Sade et al., 2010). Rice *OsPIP1;1* or *OsPIP2;2*-transgenic *Arabidopsis* plants showed enhanced tolerance to salt and drought stress (Guo et al., 2006). Conversely, the overexpression of *VvPIP2;4N* in grapevine plants resulted in good growth under favorable water conditions, but more severe damage than the control plants under drought stress (Perrone et al., 2012). Overexpression of a barley AQP *HvPIP2;1*, the expression of which was down-regulated under salt stress, could increase salt sensitivity in transgenic rice plants (Katsuhara et al., 2003). Our previous study on ectopic soybean overexpression of *VzPIP2;1* from drought-tolerant vetiver grass suggested that gene expression mediated by *A. tumefaciens* transformation at the whole plant level decreased the drought resistance of transgenic soybean plants, while the composite soybean plants with transgenic hairy roots mediated by *A. rhizogenes* showed enhanced drought tolerance (Hu et al., 2016). Thus, the response of plant *PIPs* to abiotic stresses such as salinity, drought, and cold are complex, and homologously overexpressed or heterologously expressed *PIPs* may either beneficially or adversely effect stress tolerance depending on the investigated AQP genes, organs, or plant species or stress factors (Maurel et al., 2008).

Tonoplast intrinsic proteins, as a major and plant-specific subfamily of AQPs, can regulate water exchange between the cytoplasm and vacuole and control cell water homeostasis involved in cellular storage, osmotic adjustment and turgor control, cell signaling and digestion, and abiotic stress tolerance (Peng et al., 2007; Wang et al., 2011; Xin et al., 2014; Song et al., 2016). Therefore, plants or crops may be conferred with increased tolerance toward different abiotic stresses through a suitable *TIP*-transgenic approach (Srivastava et al., 2014). For example, ectopic overexpression of *TsTIP1;2* from the halophyte *Thellungiella salsuginea* in *Arabidopsis* notably increased tolerance for drought, salt, and oxidative stresses (Wang et al., 2014). Transgenic *Arabidopsis* plants expressing *SlTIP2;2* from *Solanum lycopersicum* exhibited enhanced salt stress tolerance by regulating ion (including Na^+^ and K^+^) homeostasis and antioxidant enzyme activities under salt treatment (Xin et al., 2014). A high water channel activity was displayed by *PgTIP1-*heterologous expression in *Xenopus laevis* oocytes and in yeast, and a significant role in growth and development under favorable conditions and enhanced salt and drought tolerance, together with upregulated expression of the stress-related genes, was demonstrated in *PgTIP1-*transgenic *Arabidopsis* plants (Lin et al., 2007; Peng et al., 2007; Li and Cai, 2015). In our work, we successfully obtained *PgTIP1*-transformed soybean cotyledon hairy roots and composite plants mediated by *A. rhizogenes*, and *PgTIP1*-transgenic plants (lines N and J11) through the pollen tube pathway without a marker gene (**Figures 1A**, **2A**, **4B**). However, as a technical problem occurred as a result of the large and complex soybean genome and sharing of exogenous *PgTIP1* with high homology of soybean *GmTIPs* family itself, we thus adopted nested PCR (with 0.005% sensitivity) instead of standard PCR (with 0.5% sensitivity; Ao et al., 2011) for positive identification of *PgTIP1-*transformed soybean materials, and showed fine effectiveness (**Figures 1A-c**, **2B**, **4A**).

Under salt stress, and compared to empty vector-transformed cotyledon hairy roots or composite plants, the *PgTIP1*-transformed cotyledon hairy roots or composite plants suffered far lighter salt injury symptoms, and this could be well reflected from the biomass reduction (fresh and dry weight, or maximal length of hairy roots) (**Figures 1B,C**, **2C**) or withering degree (**Figure 2A-c**), leaf RWC drop and REL rise (**Figure 2D**) of composite plants. Of course, more important explanations for the differences in salt injury between *PgTIP1*-transformed and empty vector-transformed cotyledon hairy roots or composite plants exist in that Na^+^ contents in *PgTIP1*-transformed cotyledon hairy roots or roots, stems, and leaves of *PgTIP1*-transformed composite plants lowered significantly under salt stress in comparison with the empty vector-transformed, while K^+^ levels were relatively better maintained at the same time, eventually leading to notably lower Na^+^/K^+^ ratios than the empty vector-transformed (**Figures 1D,E**, **3A–C**). This is the first study on the aspect of root-specificity and heterologous overexpression of *PgTIP1* that demonstrated that water relations and ion homeostasis of *PgTIP1*-transformed soybean cotyledon hairy roots or composite plants are well adjusted under salt stress, and thus confer enhanced salt tolerance. On this basis, our further investigation using *PgTIP1*-transgenic soybean lines (N and J11) showed that at the whole plant level, apart from a similar ability to adjust water relations (**Figure 4C**) as well as less salt damage (expressed as a REL) (**Figure 4E**) as the above-mentioned soybean hairy roots test, plant root vigor (**Figure 4D**) and a series of photosynthesis and fluorescence parameters, including leaf chlorophyll and carotenoid contents, chla/b ratio, and Pn, Tr, and Fv/Fm values (**Figure 5**), were well maintained under salt stress in comparison to WT. With regards to Na^+^ and K^+^ contents and Na^+^/K^+^ ratio, together with the Cl^-^ contents in the roots, stems, and leaves of salt-stressed *PgTIP1*-transgenic soybean plants exposed to salt stress, similar regulating effects were also observed as the above-mentioned *PgTIP1*-transformed soybean hairy roots test, particularly line J11 which performed best (**Figures 6A–D**). *Arabidopsis* plants overexpressing *PgTIP1* displayed faster growth and enhanced salt stress tolerance through the enhancement of tonoplast permeability and increased water movement from the cytosol to vacuole, and greater accumulation of Na^+^ due to the larger vacuole (Peng et al., 2007; Li and Cai, 2015). AQPs may participate in ion homeostasis at the whole plant level by regulating the ratio of apoplastic/symplastic water flow, thus directing solute flux through different plant tissues, and furthermore, it has been reported that AQPs together with K^+^ channels can function as plant osmo-regulators to maintain cytosolic osmolarity and increase tolerance to drought or other stresses (Wang et al., 2016). This might explain the retention of Na^+^ and Cl^-^ in the roots and maintenance of water content in the leaves, which conferred favorable regulation of water relation and ion homeostasis of *PgTIP1*-transgenic soybean plants under salt stress. In addition, oxidative damage resulting from excessive ROS is the main secondary factor, apart from osmotic and ionic stresses, influencing the plant response under saline conditions (Munns and Tester, 2008; Zhao et al., 2017). Li and Cai (2015) further showed that the leaves of *PgTIP1*-transgenic *Arabidopsis* plants accumulated less H_2_O_2_ than WT under salt stress, which suggests that *PgTIP1* transformation is also beneficial for relieving oxidative stress. In our study, when compared with WT, H_2_O_2_ contents in the roots and leaves of the salt-stressed *PgTIP1*-transgenic soybean lines N and J11 did not show the increase that WT did, but rather exhibited an obvious decrease. Additionally, $O_{2}^{•-}$ production rates rose slightly in the roots but dropped in the leaves (**Figures 7A,B**), which was consistent with enhanced activities of SOD, POD, CAT, and APX in the roots or leaves under salt stress (**Figures 7C,D**). Li and Cai (2015) suggested that the heterologous expression of *PgTIP1* led to the upregulated expression of stress-related genes, such as *CBF3, MYB15, COR47, ICE1*, etc., which could contribute to the salt tolerance of the transgenic *Arabidopsis* plant. In this work, we also found that the expression levels of *GmPOD*, *GmAPX1*, *GmSOS1*, and *GmCLC1* in the roots and leaves of *PgTIP*-transgenic lines were also reinforced in comparison to WT, but there were no differences in the expression of endogenous soybean *GmTIP1;1* and *GmTIP1;3*, which shared the highest homology with *PgTIP1* (**Figure 8**). This indicates that heterologous *PgTIP1* transformation into soybean has an obvious positive contribution toward salt tolerance at the transcriptional level of stress-related genes coding ion transporters and antioxidant enzymes. Xin et al. (2014) reported that, tomato *SlTIP2;2*-transformed *Arabidopsis* plants exhibited enhanced salt stress tolerance, which has close links with higher K^+^/Na^+^ ratio (for ion homeostasis) and increased SOD, CAT, and POD activities (for ROS scavenging) than the wild-type control under salt stress. Wang et al. (2014) also demonstrated that, *TsTIP1;2-*overexpressed *Arabidopsis* plants displayed strong tolerance against salt or oxidative stress through the capability of direct conducting H_2_O, and the indirect facilitation of Na^+^ influx or H_2_O_2_ transport into the vacuoles. We also further need to seek more evidence at the subcellular level of vacuoles in future.

## Conclusion

Firstly, the heterologous overexpression of *PgTIP1* resulted in the transformed soybean cotyledon hairy roots or composite plants mediated by A. *rhizogenes* displaying superior salt tolerance to the empty vector-transformant according to the ameliorative roles in hairy root growth reduction, leaf RWC drop, and REL rise of composite plants under salt stress. Additionally, a decline in K^+^ contents, increase in Na^+^ contents and Na^+^/K^+^ ratios in the hairy roots, stems, or leaves were effectively mitigated by *PgTIP1* transformation, especially with respect to the stems and leaves of composite soybean plants. At whole plant level, pollen-tube transformation pathway *PgTIP1*-transgenic soybean lines possessed stronger root vigor, lighter cell membrane damage to the roots and leaves, improved SOD, POD, CAT, and APX activities along with the enhanced expression of *POD* and *APX* genes under salt treatment, steadily increased values of leaf RWC, Tr, and Pn, and smaller declines in chlorophyll and carotenoid contents when exposed to salt stress compared to WT. With regards to transport or distribution of salty ions in salt-stressed *PgTIP1*-trasgenic soybean lines, the heterologous transformation of *PgTIP1* into soybean plants could readjust the distribution pattern of Na^+^, K^+^, and Cl^-^ in the roots and shoots (stems and leaves), i.e., the absorbed Na^+^ and Cl^-^ mainly accumulated in the roots so as to reduce their transport to the shoots, and the root-absorbed K^+^ was simultaneously promoted to be transported toward the shoots. This may be related to *PgTIP1* transformation- and salt stress-induced enhancement of the expression of *GmSOS1* and *GmCLC1* genes, which control Na^+^ and Cl^-^ into and out of the cell, and the transport and intracellular compartmentation of soybean plants. Finally, these results reveal that the cause of enhanced salt tolerance of heterologous *PgTIP1-*transformed soybeans lies in its positive regulation of water relations, ion homeostasis, and ROS scavenging under salt stress both at the root-specific and whole plant levels. Therefore, *PgTIP1* can be considered as a good candidate gene for the production of new salt tolerant plant germplasms and crop cultivars in the future.

## Author Contributions

JA, ZH, and HC conducted the experiments, collected and analyzed all data. BY and WC designed the experiments. BY, JA, and BC interpreted the data and wrote the manuscript. All authors read and approved the final version of the manuscript.

## Conflict of Interest Statement

The authors declare that the research was conducted in the absence of any commercial or financial relationships that could be construed as a potential conflict of interest.
